# Physical and psychological effects of a long-term supervised self-exercise program during hemodialysis in elderly dialysis patients: A single-site pilot study in a Japanese community setting

**DOI:** 10.1097/MD.0000000000038963

**Published:** 2024-07-19

**Authors:** Katsumori Takamatsu, Takuya Shike, Yudai Kaneda, Divya Bhandari, Toyoaki Sawano, Akihiko Ozaki, Masaharu Tsubokura, Hiroshi Kawaguchi

**Affiliations:** a Department of Rehabilitation, Jyoban Hospital of Tokiwa Foundation, Iwaki, Fukushima, Japan; b Department of Sports and Medical fitness Re-birth, Iwaki, Fukushima, Japan; c School of Medicine, Hokkaido University, Hokkaido, Japan; d Breast and Thyroid Center, Jyoban Hospital of Tokiwa Foundation, Iwaki, Fukushima, Japan; e Department of Surgery, Jyoban Hospital of Tokiwa Foundation, Iwaki, Fukushima, Japan; f Department of Radiation Health Management, Fukushima Medical University, Fukushima, Japan; g Department of Nephrology, Jyoban Hospital of Tokiwa Foundation, Iwaki, Fukushima, Japan.

**Keywords:** dialysis patient, exercise therapy, hemodialysis, intradialytic exercise, physical function

## Abstract

Self-exercise during hemodialysis reportedly prevents functional decline. This study aimed to assess the effects of exercise on physical function during hemodialysis. From September 2014 to March 2018, 35 elderly dialysis patients participated in an exercise program 3 times a week for 24 weeks during hemodialysis under staff supervision. The Short Physical Performance Battery and muscle strength test were used to measure physical function, and the Short Form Version 2 and Self-Rating Questionnaire for Depression were used to measure psychological function. For Short Physical Performance Battery, walking speed and standing time improved significantly. Other significant improvements were observed in both knee extension muscle strength and right side of grip strength. There was also an improving trend in both Short Form Version 2 and Self-Rating Questionnaire for Depression after the intervention compared with the baseline. A long-term supervised self-exercise program during dialysis led to maintenance and improvement of physical and psychological functioning in elderly dialysis patients.

## 1. Introduction

Chronic kidney disease (CKD) is a global health issue with an estimated 697.5 million cases in 2017. Among them, 5.3 to 10.5 million people require dialysis treatment or kidney transplantation.^[1]^ Furthermore, a recent study reported that the global prevalence of all-stage CKD among adults aged 20 and older was 10.4% for men and 11.8% for women respectively.^[2]^ While hemodialysis can improve the mortality rate in CKD patients, many complications are difficult to control and prevent, particularly in the long run, such as deterioration of physical and mental function.^[3,4]^ However, exercise training has shown to enhance overall physical function, psychological well-being, and health-related quality of life (QOL) of CKD patients receiving hemodialysis.^[5,6]^

European Best Practice Guideline (EBPR) on Nutrition published in 2007 by the European Renal Association-European Dialysis and Transplant Association has explicitly highlighted the importance of exercise for patients receiving hemodialysis.^[7]^ On the other hand, in Japan, the Japanese Society of Renal Rehabilitation established the Clinical Practice Guideline for renal rehabilitation in 2018, which was the world’s first guideline solely dedicated to the renal rehabilitation exercise (RRE) program.^[8]^ This guideline provides a framework for managing long-term CKD patients treated with hemodialysis, including exercise, diet, and fluid management, medication, and education. It emphasized the importance of patient education and psychological support in engaging CKD patients in the RRE program.^[8]^ Following the guideline’s establishment, the Japanese society of renal rehabilitation then formulated a registered instructor of renal rehabilitation system to certify those who obtained the necessary skills for comprehensive RRE.^[9]^

In Japan, there has been an increasing implementation of the RRE program.^[10,11]^ However, limited information is still available regarding the physical and psychological effects of these programs on elderly hemodialysis patients during hemodialysis sessions, particularly in the long term. At this stage, we believe that it is important to conduct and report an even small sized study to strengthen evidence to address this issue. Therefore, the purpose of this study was to examine the effects of an exercise program on physical and psychological functioning in elderly hemodialysis patients undergoing hemodialysis.

## 2. Methods

### 2.1. Study design and participants

A single-center, retrospective observational pretest-posttest study design was used to investigate the effect of the RRE program on body composition, physical function, and the QOL of hemodialysis patients with CKD. All participants who underwent hemodialysis in A Hospital, located in Iwaki City, Fukushima Prefecture, Japan, between September 2014 and August 2019 were included in the study. The study included individuals who requested exercise from dialysis patients at the hospital. All patients making such requests were included, regardless of whether they had physical disabilities. Eligible patients underwent evaluations by both a nephrologist and a cardiologist to ensure that they were not at risk for morbidity, including acute illness, prior to initiating the exercise program. A Hospital is a general hospital that is particularly specialized in treating urological disorders. Overall, 148 dialysis beds are available, which is one of the largest numbers among general hospitals in the area. A Hospital’s Dialysis Center treats approximately 202 outpatients per day, and the cumulative number of monthly dialysis sessions was approximately 5868 in 2014. In 2014, Hospital A launched a kidney rehabilitation exercise program for elderly hemodialysis patients as part of its medical services, with a team of healthcare professionals. The medical team involved in the renal rehabilitation program were, nephrologist, cardiologist, nurse, physical therapist, clinical engineer, dietitian, and caregiver. Before the intervention, physicians reviewed the medical records and performed clinical examinations of all participants in the RRE program.

### 2.2. Intervention

**Figure 1.**
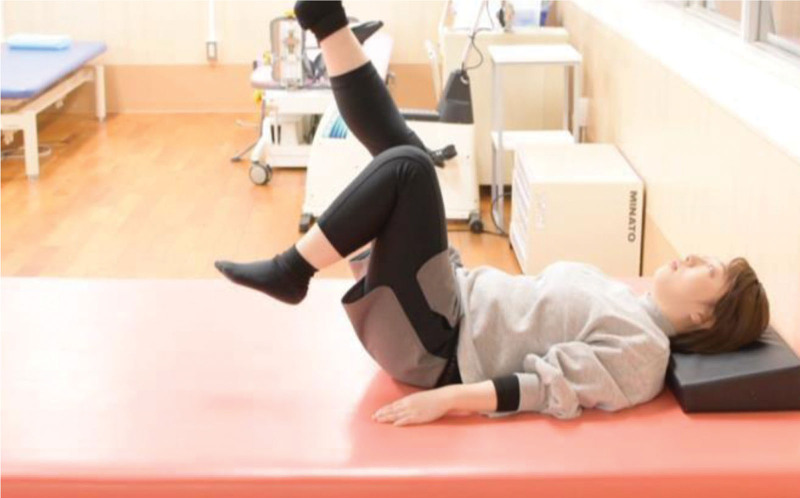
Lower Extremity Exercise 1. Strengthening of the knee and hip muscles through bilateral leg lifts with knee flexion. Figure shows the staff of this hospital.

**Figure 2.**
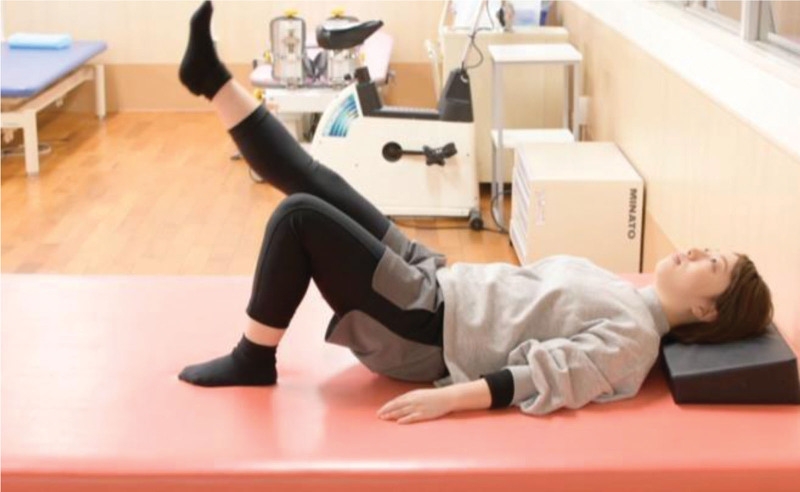
Lower Extremity Exercise 2. Strengthening of the hip and knee joint muscles via unilateral straight leg raises. Figure shows the staff of this hospital.

**Figure 3.**
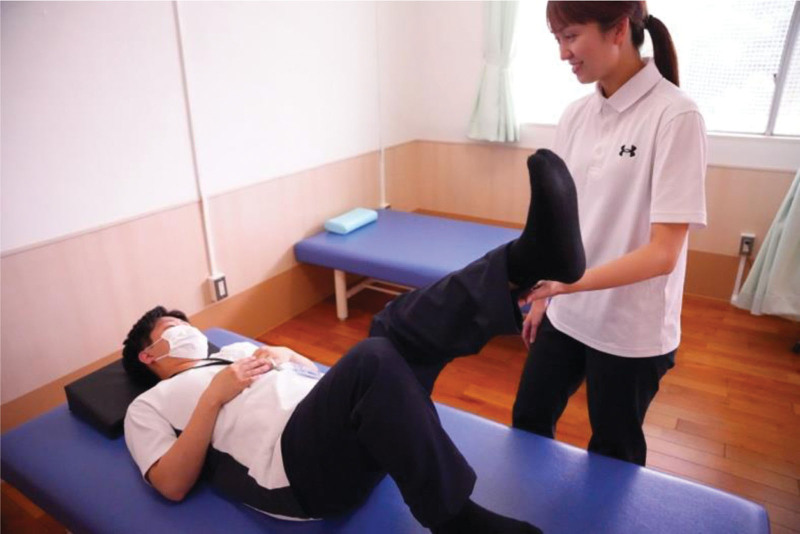
Instructional Interaction. Rehabilitation staff providing appropriate exercise instructions. All individuals depicted in the figure are staff members of this hospital.

The RRE program was developed by physical therapists, nephrologist, and cardiologist at Hospital A based on the guidelines of the American College of Sports Medicine (ACSM), taking into account factors such as frequency, intensity, time, and type.^[12]^ Regarding the exercise load, primary consideration was given to the rating of perceived exertion (RPE), with the value set to 13 or less, indicating an exercise intensity below moderate. Additionally, the exercise content was tailored to be below 40% to 60% (0.4 k‐0.6 k) of the maximal heart rate, as per the Karvonen equation. In this context, the program was intentionally structured as a moderate-intensity exercise regimen.^[13]^ Since the program content remained consistent for all subjects, individual considerations were made, including the application of dead weights on the lower limbs and incorporating intervals of rest within the program. Patients with insufficient load were provided with additional weights (0.5 kg or 0.75 kg), attached to the lower legs during the exercise. The exercise period was 24 weeks (6 months). The in-hospital exercise program was initiated 1 hour after the initiation of hemodialysis as patients’ circulatory dynamics became unstable immediately after the initiation of hemodialysis, and their blood pressure was easily lowered in the latter half of the session. The in-hospital RRE program consisted of weight-based exercise training focused on the movement of the ankle, knee, hip, and trunk joints. The exercises included flexion, extension, abduction, adduction, and rotation of the hip joint. For the knee joint, we mainly performed extension exercises. The goal is to maintain or increase muscle strength and skeletal muscle mass. The program was delivered for 40 minutes, including 1 to 2 minutes long short intervals. Participants with preexisting lumber back pain or disorder received individual instruction to control the exercise intensity and load. The patients’ blood pressures and pulse rates were measured 3 times before, during, and after the exercise. Furthermore, all participants were provided with visual instruction using digital video discs to encourage patients’ home exercise routine. The following images are excerpted from our original video of the actual exercise. In addition, exercise programs were developed to allow for supervised self-exercise. Figure 1 Lower extremity exercises, Figure 2 Lower extremity exercises, and Figure 3 Instruction.

We commenced incorporating exercise during dialysis prior to the publication of the Renal Rehabilitation Guidelines in 2018.^[6]^ Consequently, determining the appropriate timing for safe implementation was challenging. In the case of in-hospital exercises, activity initiation was set at 1 hour after the commencement of hemodialysis. This decision was informed by the observation that patients’ hemodynamics often exhibit instability immediately after the initiation of hemodialysis, with blood pressure decreasing in the latter half of the day.^[14]^ We scheduled the physical activity to conclude within the first 2 hours of the hemodialysis session.

### 2.3. Outcomes

Blood pressure and pulse were measured 3 times at the beginning, middle, and end of the exercise, and the RPE was recorded after the exercise. The ACSM guideline and the Anderson criteria were used to reference risk management at the beginning and during exercise.^[12]^ During exercise therapy, the patients were monitored by a multidisciplinary team (such as nurses, dietitians, clinical engineers, and physical therapists). In addition, nurses conducted questionnaire surveys and provided feedback on the results, dietitians conducted dietary interviews, measured body composition and provided feedback on the results, clinical engineers responded to alarms of dialysis equipment and checked for abnormalities at the puncture site, and physical therapists checked for accuracy of exercise, pain, and evaluated physical functions and provided feedback on the results. In addition, a monthly meeting on exercise therapy during dialysis was held to share information among the multidisciplinary staff. The objective of the meeting was to understand the current status of patients, discuss matters related to the introduction of new patients, and share information among satellite facilities introducing exercise therapy.

### 2.4. Data collection

Data on the subjects were extracted retrospectively from their medical records. Specifically, data on the following clinical factors were extracted: the name of the main disease that led to end-stage renal failure, age at the start of the study, weight at the start of exercise and 6 months later, the cardio-thoracic ratio values, albumin, total protein, blood urea nitrogen before and after dialysis, creatinine and C-reactive protein before and after dialysis, and body composition measurements: muscle mass, body fat, and skeletal muscle mass index (SMI) were extracted. Age was averaged at the start of exercise, and body weight was averaged from the date of the start of exercise and 6 months later. For the cardio-thoracic ratio values, the shortest date from the start date of the exercise was used. And for the data after 6 months from the start of exercise, the shortest date from after 6 months was used.

For blood test values (C-reactive protein, albumin, total protein, blood urea nitrogen, and creatinine before and after dialysis), the shortest data from the start date of exercise were used for values after 6 months.

Short Physical Performance Battery (SPPB), isometric knee extension muscle strength, grip strength, and Barthel Index were evaluated to measure motor function. The SPPB was divided into 3 tests: a standing test, a gait test, and a chair stand test. For SPPB, there is a cutoff value to determine the level of physical function according to the score: 0 to 6 points are considered low function, 7 to 9 points are considered medium function, and 10 to 12 points are considered high function.^[15,16]^

For the measurement of motor function, data within 1 week from the date of exercise initiation and data within 1 week from 6 months after the start of exercise were used.

Similarly, for mental function, we used the Short Form Version 2 (SF36v2) Japanese version of health-related QOL and the Self-Rating Questionnaire For Depression.^[17]^ The SF36v2 measures 8 health concepts and assesses health-related QOL (Table 1).

**Table 1:** Eight subscales of the SF36.^[18]^

Subscales	Abbreviation	Interpretation	
		Lowcase	Highcase
Physical functioning	PF	High difficulty in performing ADL activities on their own.	Capable of performing many activities as well as high-mobility activities.
Physical role	PR	Physical-related problems with activity in the past month.	No physical-related problems with activity in the past month.
Bodily pain	BP	Intense physical pain in the past month that has severely interfered with their activities.	No physical pain in the past month and activity was not hampered.
General health	GH	Health gradually deteriorated.	Good health condition.
Vitality	VT	Feeling constantly fatigued for the past month.	Always full of vitality during the past month.
Social functioning	SF	Severely disturbed by a physical or psychological event in the past month with family, friends, or others.	No physical or psychological events interfered in the past month with socializing with family, friends, or others.
Emotional role	ER	Had psychological problems during daily activities in the past month.	No psychological problems during daily activities in the past month.
Mental health	MH	Constantly depressed in the past month.	State of being calm, not depressed, during the past month.

Descriptions of the 8 measures as represented by their numerical values are given in the table below; each of the 8 measures assesses a physical functioning aspect, a mental health aspect, a physical component summary, a mental component summary, a role, and a social component summary. There are 3 other component scoring methods for the physical, mental, role, and social dimensions that can be represented by the 8 measures.^[15]^ There are three other component scoring methods for the physical, mental, role, and social dimensions that can be represented by the eight measures. These are referred to as the “physical component summary (PCS)” for the physical dimension, the “mental component summary (MCS)” for the mental dimension, and the “role social component summary (RCS)” for the role and social dimensions. Data within the first week after the start of exercise and the shortest data of 6 months after the start of exercise were used. The shortest data were used.

SPPB is based on the definition of sarcopenia and the cutoff value (<8 points) of the revised version of the European Working Group on Sarcopenia in Older People.^[19]^ If the score is below the cutoff value, there is a high possibility of sarcopenia, and it is also considered to be a predictor of falls. Isometric knee extension muscle strength was measured in the chair sitting position with the hip and knee joints fixed at 90° using a handheld dynamometer (Sakai Medical Co., Ltd., mobie MT100) and an optional pull sensor MT150.

### 2.5. Statistical analysis

In this study, 2 analyses were conducted: first, to clarify the effects of pre- and post-exercise on motor function, corresponding parametric *t* tests were used for the balance test, walking test, and standing test; non-parametric tests were used for the SPPB point, handgrip, and knee extension. For the SPPB point, handgrip, and knee extension, the non-parametric Wilcoxon signed rank sum test was used for analysis.

Second, to clarify the effects of pre- and post-exercise on mental and functional aspects, we used the corresponding parametric *t* tests for the componential summary scores physical component summary, mental component summary, role and social component summary of the health-related quality of life (QOL) SF36v2 and the Toho University depression test, Self-Rating Questionnaire for Depression. To evaluate the health-related QOL SF36v2 (physical functioning, daily role functioning, body pain, overall sense of well-being, vitality, and social functioning), we used the Wilcoxon signed rank sum test, a non-parametric test. The significance level for these was set at 5%. Stata/IC 15.0 was used for all data analyses.

### 2.6. Ethical approval

This study was reviewed and approved by the Ethics Committee of the Tokiwa Foundation (approval number: JHTF-2020–028). This exercise intervention was conducted as part of routine clinical practice, supported by a substantial body of evidence, after obtaining informed consent from participants for the intervention. Subsequent analysis was performed retrospectively using data extracted from medical records. Additional consent for this analysis was deemed unnecessary, as the ethics board approved the study under these conditions.

## 3. Results

In this study, a total of 37 hemodialysis patients enrolled in the renal rehabilitation exercise program and had participated in the program for more than 6 months. Of these patients, 1 died of cardiovascular diseases, and 1 decided to quit joining the program for personal reasons. Excluding these 2 individuals, 35 patients were considered in the following analyses. The mean age of the participants was 72.0 (SE 2.0) years, and 54% of them were male. The mean duration of hemodialysis for participants was an average of 10.7 (SE 2.3) years. The major primary diseases that led to end-stage renal failure were diabetic kidney disease (31%), nephrosclerosis (31%), chronic glomerulonephritis (14%), IgA nephropathy (9%).

The patients’ characteristics at the baseline and after the intervention are shown in Table 2. Skeletal muscle mass, SMI, blood urea nitrogen, body fat, and estimated glomerular filtration rate were patient background factors that showed significant differences after the exercise intervention compared to baseline. There was no significant difference at the baseline and after the intervention in the remaining variables.

**Table 2:** Patients’ characteristics.

Variable name	Base levels (N = 35*)	After the intervention (N = 35*)	*P*-value
Primary diagnosis			
Diabetic kidney disease	11 (31%)		
Nephrosclerosis	11 (31%)		
Chronic itchy globe nephritis	5 (14%)		
IgA nephrosis	3 (9%)		
Other	2 (6%)		
Unknow cause	3 (9%)		
Age (year)	72.0 (SE 2.0)		
Sex			
Men	19 (54%)		
Women	16 (46%)		
Weight (kg)	53.6 (SE 1.7)	52.6 (SE 1.7)	.17
Albumin (g/dL)	3.62 (SE 0.1)	3.62 (SE 0.1)	.73
Total protein (g/dL)	6.57 (SE 0.1)	6.51 (SE 0.1)	.53
Blood urea nitrogen (mg/dL)			
Before hemodialysis	65.02 (SE 3.6)	57.18 (SE 2.9)	.04
After hemodialysis	17.16 (SE 1.4)	13.28 (SE1.1)	.004
Creatinine (mg/dL)			
Before hemodialysis	9.8 (SE 0.8)	9.48 (SE 0.7)	.77
After hemodialysis	3.4 (SE 0.39)	3.1 (SE 0.32)	.09
C-reactive protein (mg/dL)	0.82 (SE 0.4)	0.3 (SE 0.1)	.66
Muscle mass (kg)	20.3 (SE 0.8)	21.1 (SE 0.8)	.004
Body fat (kg)	15.5 (SE 1.2)	13.7 (SE 1.1)	<.001
Skeletal muscle mass index (kg/m^2^)	6.47 (SE 0.2)	6.63 (SE 0.2)	.05
Cardio-thoracic ratio (%)	52.2 (SE 1.2)	52.6 (SE 0.9)	.33
Estimated glomerular filtration rate (%)			
Before hemodialysis	4.44 (SE 0.31)	4.54 (SE 0.36)	.27
After hemodialysis	15.5 (SE 1.47)	17.3 (SE 1.65)	.03

* Analysis of the number of patients excluding those for whom data are not available; SE = standard error.

The physical functions at the baseline and after the intervention are shown in Table 3. There was a significant improvement in SPPB after the intervention compared with the baseline (10.2 (SE 0.3) vs 9.6 (SE 0.3), *P* < .02). On the SPPB subscales, balance test, gait speed, and standing and sitting time were significantly improved compared to baseline and post-intervention. Other muscle strength and RPE showed predominantly reduced fatigue and increased muscle strength compared to baseline.

**Table 3:** Physical function before and after intervention among the participants.

Variable name	Baselevels (N = 33*)	After the intervention (N = 33*)	*P*-value
Short Physical Performance Battery	9.6 (SE 0.3)	10.2 (SE 0.3)	.02
** **Balance test	3.4 (SE 0.2)	3.6 (SE 0.2)	.08
** **4.0-m gait speed (m/s)	0.8 (SE 0.3)	0.9 (SE 0.3)	<.001
Chair stand test (s)	12.2 (SE 0.9)	11.2 (SE 0.6)	.04
Hand grip (right side, kg)	20.9 (SE 1.4)	22.0 (SE 1.4)	<.001
Hand grip (left side, kg)	19.7 (SE 1.3)	20.4 (SE 1.4)	.24
Knee extension (right side, kg)	19 (SE 1.1)	21.6 (SE 1.4)	<.001
Knee extension (left side, kg)	17.7 (SE 1.1)	20.0 (SE 1.3)	<.001
Rate of perceived exertion	12.05 (SE 0.2)	11.57 (SE 0.2)	<.001

* Analyzed among the 33 participants the data of which is available.

In summary, the results of physical function 6 months after the start of the exercise indicated that the patients were highly functional.

The psychological functions before and after the intervention are shown in Table 4.

**Table 4:** Psychological function before and after intervention among the participants.

Variable name	Baselevels (N = 17*)	After the intervention (N = 17*)	*P*-value
^Short Form-36 version 2^ *			
^ Physical role^	^40.5 (SE 3.4)^	^41.1 (SE 4.0)^	^.85^
^ General health^	^45.3 (SE 3.2)^	^52.9 (SE 2.6)^	^.09^
^ Bodily pain^	^46.6 (SE 2.9)^	^54.8 (SE 1.3)^	^.1^
^ Vitality^	^49.3 (SE 3.7)^	^48.8 (SE 3.4)^	^.92^
^ Social functioning^	^43.5 (SE 3.4)^	^50.9 (SE 2.8)^	^.92^
^ Emotional role^	^37.9 (SE 3.5)^	^47.4 (SE 4.0)^	^.42^
^ Physical functioning^	^36.6 (SE 4.3)^	^41.3 (SE 3.3)^	^.1^
^ Mental health^	^52.9 (SE 2.2)^	^52.0 (SE 2.8)^	^.53^
^ Physical component summary^	^36.7 (SE 4.0)^	^43.0 (SE 2.8)^	^.09^
^ Mental component summary^	^54.6 (SE 2.7)^	^55.5 (SE 2.2)^	^.8^
^ Role/Social component summary^	^46.3 (SE 2.7)^	^43.0 (SE 4.3)^	^.37^
^Self-Rating Questionnaire for Depression^	^6.1 (SE 0.2)^	^6.2 (SE 0.2)^	^.73^

* Statistics on 17 people, excluding the criteria.

The SF36v2 tended to improve post-intervention compared to baseline, although there were no statistically significant changes compared to baseline on all 8 scales.

Table S1, Supplemental Digital Content, http://links.lww.com/MD/N228. Physical function before and after intervention among the participants (gender differences).

The findings from our study suggest that muscle strength shows a more significant improvement in men, whereas physical performance, encompassing walking, standing, and balance abilities, exhibits a more significant improvement in women.

Table S2, Supplemental Digital Content, http://links.lww.com/MD/N229. Psychological function before and after intervention among the participants (gender differences).

Comparisons of gender differences are also shown in Table S2, Supplemental Digital Content, http://links.lww.com/MD/N229; no significant differences were found for any of the scales except general health.

## 4. Discussion

This study evaluated the effects of a 6-month exercise therapy intervention during hemodialysis on physical and psychological functioning in elderly hemodialysis patients. The results showed an improved physical and psychological functioning after the intervention compared with baseline. In particular, both muscle strength and physical performance were improved. In this study, patients with end-stage renal failure were more likely to have diabetes mellitus. In addition, a comparison of baseline and post-exercise results showed that in addition to increases in muscle strength and skeletal muscle mass, post-dialysis blood urea nitrogen decreased, presumably due to increased blood flow to skeletal muscle, thereby facilitating solute removal and improving dialysis efficiency. The lack of change in total protein or albumin suggested that this decrease may have been due to exercise during dialysis rather than a decrease in nutritional status. One previous study also reported that exercise during dialysis improves dialysis efficiency.^[20]^

The findings regarding physical function in the current study align with those of prior research.^[21–23]^ Renal complications are a very common consequence of diabetes, with an estimated prevalence of approximately 42% in Japan. Similarly, in our cohort, 31% of the kidney conditions are primarily caused by diabetes. In this respect, it seems improbable that the presence of diabetes would have an impact on the enhancement of physical function as studies have consistently demonstrated the significant effectiveness of exercise in improving functional outcomes for individuals with diabetes. However, these previous studies were conducted in a relatively young age group, and there are few reports of studies on dialysis patients with an average age of over 70 years. In the past surveys, factors that led to favorable results included the ability to exercise continuously (low-intensity resistance exercise) on dialysis days and the reduction of bed rest time due to dialysis when exercise was introduced.^[24,25]^ Considering this background, there are two additional reasons for such positive results that require further scrutiny.

The first reason is that we could continuously implement strength training during the resting time of dialysis. It has been reported that the exercise tolerance of hemodialysis patients is almost similar to that of patients with heart failure or chronic obstructive pulmonary disease.^[26]^ There is a concern that muscle atrophy and deterioration of the nervous system due to rest and bed rest may occur, especially in the elderly undergoing hemodialysis.^[26]^ In addition to decreased exercise tolerance, decreased walking ability is observed and decreased walking ability leads to decreased ADL, which affects home life, instrumental ADL, and outpatient dialysis visits. Since hemodialysis is performed 3 times a week (12 times a month) and patients are forced to rest for 3 to 4 hours at a time (36–48 h a month), it is thought that the introduction of exercise therapy may reduce the amount of time spent in bed at rest, which may ultimately contribute to the improvement of physical functions.

The second reason is that proper guidance by a team of multidiciplinary health care professionals may have facilitated the proper implementation of the exercise program. In addition, because the intervention was delivered by the team, it may have improved the quality of exercise and resulted in functional improvements. Since there is no evidence for the appropriate exercise load during dialysis, referring to the ACSM guideline and Anderson criteria, we set an exercise load with a maximum RPE of 13, which is expected to be effective for muscle strengthening. Evidently, in our study, the quality of the exercise was improved by setting the exercise load to that specific level and by monitoring the participants as needed to check for any discrepancy in methods. In the case of the elderly, it has been suggested that skeletal muscle mass and exercise function can be improved by increasing the frequency of exercise even when the intensity of exercise is low. Accordingly, in our study, the exercise load was set at a low to moderate level with an RPE of 13 or less, and the frequency of exercise was increased. The increase in the frequency of exercise may have, in turn, increased SMI. Increasing muscle mass improves physical function as well as the ability to exercise better.

Previous studies have also highlighted the numerous benefits of collaborative team-based care, including functional improvement and significant improvement in health-related outcomes.^[27,28]^ Similar results were noted among the elderly population as well.^[29]^

We also investigated gender differences in both physical and mental functioning as supplementary information. Previous studies on gender disparities have indicated that grip strength and muscle strength tend to be lower in women.^[30]^ However, there is limited understanding of differences in physical performance, excluding muscle strength, and the rate of improvement with exercise among the elderly. The findings from our study suggest that muscle strength shows a more significant improvement in men, whereas physical performance, encompassing walking, standing, and balance abilities, exhibits a more significant improvement in women. Possible explanations for these findings could include the decrease in walking and standing speed being linked to reduced activity, with women representing a larger proportion of engagement in daily hobbies and social activities among the elderly, potentially influencing these improvements. Additionally, as mentioned earlier, men generally have higher muscle mass and strength on average compared to women, which may have contributed to the observed enhancement in muscle strength through exercise.

As for the results of psychological functioning in this study, there was no significant difference in all items, which is similar to the results of previous studies. The analysis was conducted separately for men and women, but no significant differences were found compared to the overall population. Changes in GH (General health) were observed only in males, but there were no other significant differences. As a result, there were no marked differences overall, but a trend toward maintenance was observed. In fact, So Yon Rhee et al reported no significant differences in all other items except for 1 item of mental functioning SF36v2 and did not find any decline in psychological functioning as in the present study. Continuing exercise therapy during dialysis can result in the prevention of deterioration in psychological function.

It has been reported that patients with chronic renal failure and those on dialysis may experience anxiety and depression to the same extent, and that prolonged dialysis may lead to a decrease in QOL and self-efficacy.^[31–33]^ Our results suggest that exercising during hemodialysis may help maintain patients’ psychological function and solve these psychological problems.

Nonetheless, more comprehensive studies should be conducted in the future to thoroughly evaluate the effectiveness of this program and to examine the effects of exercise on patients who plan to undergo renal transplantation, as more and more patients have been considered for renal transplantation in recent years in addition to those who plan to undergo dialysis. While clear guidelines for exercise therapy for patients under 70 years of age considering kidney transplantation are currently lacking, we believe that incorporating exercise into the care of chronic kidney disease patients scheduled for transplantation may be a relatively safe approach. This belief is grounded in the observation that elderly dialysis patients, who often have numerous comorbidities, engage in safe and regular exercise during dialysis, experience no adverse post-exercise conditions, and engage in low-intensity exercise below an RPE of 13, which has an extremely low impact on renal function. Additionally, the physical frailty of prospective kidney transplant recipients may potentially negatively impact post-transplantation recovery and immune function. We are of the opinion that these factors collectively support the implementation of an exercise program as a preventive measure against frailty in individuals considering a kidney transplant.^[34]^

## 5. Limitation

Although this is one of the few studies reported in Japan that evaluated the effects of self-training on physical and mental function in elderly patients undergoing hemodialysis, it should be noted that there are certain limitations. For the current research paper, we did not set a control group and we cannot say for sure that the observed changes after the exercise was caused by the exercise. Exercise during hemodialysis is based on supervised self-exercise, and patients with high ADL levels are given priority. In addition, exercise is performed simultaneously at the same time of day, which causes time constraints and needs to be performed in a limited amount of time, and we believe this might have caused a selective bias. It is necessary to consider exercise programs and intervention methods for patients with significant muscle weakness and low physical activity. It may be necessary to expand further the number of patients who exercise during dialysis and follow the changes in physical and psychological functions through long-term continuation.

## 6. Conclusion

The pilot study delved into the impact of a prolonged supervised self-exercise resistance program during hemodialysis on elderly patients, uncovering improved physical and functional outcomes. For the current research paper, we did not set a control group and we cannot say for sure that the observed changes after the exercise was caused by the exercise. However, the exercise-induced improvements in psychological well-being did not reach statistical significance. These results highlight the importance of medical team engagement and initiatives like these as vital interventions for elderly patients undergoing hemodialysis. Additionally, it is advisable to conduct more comprehensive research to thoroughly evaluate the effectiveness of this program in the times ahead.

## Author contributions

**Data curation:** Katsumori Takamatsu, Akihiko Ozaki.

**Formal analysis:** Katsumori Takamatsu.

**Writing – original draft:** Katsumori Takamatsu.

**Writing – review & editing:** Takuya Shike, Yudai Kaneda, Divya Bhandari, Toyoaki Sawano, Akihiko Ozaki, Masaharu Tsubokura, Hiroshi Kawaguchi.

## Supplementary Material




