# Supplementary material for: Physical and psychological effects of a long-term supervised self-exercise program during hemodialysis in elderly dialysis patients: A single-site pilot study in a Japanese community setting
Source: Medicine (Baltimore). 2024 Jul 19;103(29):e38963. doi: 10.1097/MD.0000000000038963 (PMC11398778; doi:10.1097/MD.0000000000038963)
Supplement: Supplementary file 1 [file medi-103-e38963-s001.docx]

Supplementary Material 1. Physical function before and after intervention among the participants (Gender Differences)

| **Variable name** | **Baselevels (^†^)** | **After the intervention (^†^)** | **P-value** |
| --- | --- | --- | --- |
| **Short Physical Performance Battery** |  |  |  |
| **Male** | 9.50(SE 0.6) | 9.78(SE 0.5) | 0.1 |
| **Female** | 9.47(SE 0.5) | 9.71(SE 0.5) | 0.02 |
| **Balance test** |  |  |  |
| **Male** | 3.33 (SE 0.2) | 3.50(SE 0.2) | 0.09 |
| **Female** | 13.48 (SE 0.2) | 11.31(SE 0.2) | 0.08 |
| **4.0-meter gait speed (m/sec)** |  |  |  |
| **Male** | 0.80 (SE 0.4) | 0.82(SE 0.4) | 0.07 |
| **Female** | 0.82 (SE 0.3) | 0.91(SE 0.3) | 0.002 |
| **Chair stand test (sec)** |  |  |  |
| **Male** | 11.43 (SE 0.8) | 11.34(SE 0.6) | 0.4 |
| **Female** | 13.48 (SE 1.7) | 11.31(0.9) | 0.01 |
| **Hand grip (right side, kg)** |  |  |  |
| **Male** | 13.42 (SE 1.7) | 14.27 (SE 1.8) | <0.001 |
| **Female** | 14.46 (SE 2.0) | 15.45 (SE 2.0) | 0.06 |
| **Hand grip (left side, kg)** |  |  |  |
| **Male** | 15.66 (SE 1.6) | 16.81 (SE 1.7) | 0.04 |
| **Female** | 16.19 (SE 1.9) | 17.49 (SE 2.0) | 0.6 |
| **Knee extension (right side, kg)** |  |  |  |
| **Male** | 20.8 (SE 1.8) | 22.9 (SE 2.0) | 0.03 |
| **Female** | 16.9 (SE 1.0) | 19.4 (SE 1.8) | 0.02 |
| **Knee extension (left side, kg)** |  |  |  |
| **Male** | 18.25 (SE 1.5) | 21.09 (SE 1.8) | 0.002 |
| **Female** | 16.58 (SE 1.2) | 18.68 (SE 1.7) | 0.01 |
| **Rate of perceived exertion** |  |  |  |
| **Male** | 11.58 (SE 0.2) | 11.26 (SE 0.2) | 0.02 |
| **Female** | 12.63 (SE 0.3) | 11.94 (SE 0.3) | <0.001 |

^†^N=17(Mele),16(Female)
