# Supplementary material for: Physical and psychological effects of a long-term supervised self-exercise program during hemodialysis in elderly dialysis patients: A single-site pilot study in a Japanese community setting
Source: Medicine (Baltimore). 2024 Jul 19;103(29):e38963. doi: 10.1097/MD.0000000000038963 (PMC11398778; doi:10.1097/MD.0000000000038963)
Supplement: Supplementary file 2 [file medi-103-e38963-s002.docx]

Supplementary Material 2. Psychological function before and after intervention among the participants (gender differences)

| **Variable name** | **Baselevels (^†^)** | **After the intervention (^†^)** | **P-value** |
| --- | --- | --- | --- |
| **Short Form-36 version 2^†^** |  | | |
| **Physical role** |  | | |
| **Male** | 36.2 (SE 4.1) | 45.3 (SE 6.2) | 0.84 |
| **Female** | 45.3 (SE 5.3) | 49.5(SE 4.1) | 0.19 |
| **General health** |  | | |
| **Male** | 40.0 (SE 3.4) | 49.9 (SE 2.8) | 0.02 |
| **Female** | 51.1 (SE 5.0) | 55.91 (SE 4.8) | 0.66 |
| **Bodily pain** |  | | |
| **Male** | 43.4 (SE 3.3) | 42.0 (SE 3.2) | 0.09 |
| **Female** | 55.8 (SE 5.0) | 56.7(SE 3.0) | 0.82 |
| **Vitality** |  | | |
| **Male** | 53.83 (SE 4.8) | 53.16 (SE 4.4) | 0.1 |
| **Female** | 49.43 (SE 4.8) | 46.62 (SE 4.1) | 0.8 |
| **Social functioning** |  | | |
| **Male** | 41.99 (SE 5.5) | 44.85 (SE 4.3) | 0.74 |
| **Female** | 50.58 (SE 3.2) | 51.38 (SE 3.3) | 0.97 |
| **Emotional role** |  | | |
| **Male** | 40.3 (SE 3.5) | 47.8 (SE 5.1) | 0.43 |
| **Female** | 35.8 (SE 6.4) | 42.1(SE 5.0) | 0.34 |
| **Physical functioning** |  | | |
| **Male** | 28.96 (SE 5.4) | 38.19 (SE 4.5) | 0.06 |
| **Female** | 45.21 (SE 5.7) | 44.76 (SE 4.7) | 0.61 |
| **Mental health** |  | | |
| **Male** | 53.83 (SE 3.6) | 53.16 (SE 4.5) | 0.82 |
| **Female** | 48.71 (SE 2.8) | 52.0 (SE 3.4) | 0.5 |
| **Physical component summary** |  | | |
| **Male** | 37.3 (SE 6.0) | 40.1 (SE 7.4) | 0.6 |
| **Female** | 32.9 (SE 5.1) | 45.4 (SE 3.1) | 0.08 |
| **Mental component summary** |  | | |
| **Male** | 53.4 (SE 4.6) | 51.9 (SE 2.7) | 0.26 |
| **Female** | 55.6 (SE 3.4) | 58.9 (SE 3.6) | 0.15 |
| **Role/Social component summary** |  | | |
| **Male** | 45.2 (SE 3.0) | 43.2 (SE 5.1) | 0.43 |
| **Female** | 47.6 (SE 9.4) | 44 (SE 8.1) | 0.32 |
| **Self-Rating Questionnaire for Depression** |  | | |
| **Male** | 6.1 (SE 0.3) | 6.1 (SE 0.3) | 0.89 |
| **Female** | 6.1 (SE 0.3) | 6.3 (SE 0.4) | 0.65 |

^†^N=9(Mele),8(Female)
